# Person-Centered Care Reduces Psychotropic Medication Usage in Randomized, Controlled, Alzheimer’s Trial

**DOI:** 10.1093/geroni/igaf122.2225

**Published:** 2025-12-31

**Authors:** Sunnie Kenowsky, Yongzhao Shao, Qiao Zhang, Sloane Heller, Martin Sadowski, Thomas Wisniewski, Arjun Masurkar, Barry Reisberg

**Affiliations:** New York University Langone Health, NYU Grossman School of Medicine, Center for Cognitive Neurology, New York, New York, United States; New York University Langone Health, New York, New York, United States; New York University Langone Health, New York, New York, United States; Columbia University Irving Medical Center, New York, New York, United States; New York University Langone Health, New York, New York, United States; New York University Langone Health, New York, New York, United States; New York University Langone Health, New York, New York, United States; New York University Langone Health, New York, New York, United States

## Abstract

Behavioral and psychological symptoms of dementia (BPSD) are prevalent and associated with poor health outcomes. Psychotropic medications, prescribed to treat BPSD, have limited efficacy and deleterious effects including stroke and death (ncbi.nlm.nih.gov/books/NBK551552). To evaluate whether comprehensive, individualized, person-centered management (CI-PCM) could improve healthcare for persons diagnosed with moderate-to-severe Alzheimer’s disease (PAD) we conducted a 28-week and a 24-week extension trial in community-residing, 65-year and older PAD (doi.org/10.1159/000455397). Twenty PAD-carepartner dyads were randomized to receive usual community care (UCC) or CI-PCM. BPSD were assessed using the Behavioral Pathology in Alzheimer’s Disease-Frequency-Weighted Severity Scale which showed significant benefit in PAD receiving CI-PCM at 52-weeks (p = 0.01, doi:10.1002/alz.090018). BPSD and medication usage were assessed at baseline, and weeks 4, 12, 28 and 52. Differences in psychotropic medications taken were compared by calculating the percent maximum daily dose of each medication taken cumulatively throughout each observation period and examining the ratio of daily medication usage in each group using the Wilcoxon Rank Sum Test. At baseline, PAD receiving CI-PCM consumed significantly less psychotropic medication than those receiving UCC (p = 0.017, doi.org/10.1002/alz.056048). Therefore, we analyzed the magnitude of daily medication used in each group on a per day average from weeks 4 to 52, compared to baseline. PAD receiving CI-PCM consumed significantly less medication on a per day average (p = 0.002) and PAD receiving UCC consumed significantly higher total amounts of psychotropic medication (p < 0.0001) from weeks 4 to 52, relative to baseline. CI-PCM is readily implemented, reduces BPSD, reduces psychotropic medication usage, and improves PAD health outcomes.

